# Needs and concerns of transgender individuals regarding interdisciplinary transgender healthcare: A non-clinical online survey

**DOI:** 10.1371/journal.pone.0183014

**Published:** 2017-08-28

**Authors:** Jana Eyssel, Andreas Koehler, Arne Dekker, Susanne Sehner, Timo O. Nieder

**Affiliations:** 1 Department for Sex Research and Forensic Psychiatry, Interdisciplinary Transgender Healthcare Centre Hamburg, University Medical Centre Hamburg-Eppendorf, Hamburg, Germany; 2 Centre for Experimental Medicine, University Medical Centre Hamburg-Eppendorf, Hamburg, Germany; University of South Australia, AUSTRALIA

## Abstract

This study investigates the needs and concerns transgender (short: trans) individuals have concerning trans healthcare (THC) in interdisciplinary THC centres. Trans individuals’ gender does not (fully/constantly) match their sex assigned at birth. To be able to live in their gender role and to prevent or minimise gender dysphoria, they might require a multidisciplinary set of transition related healthcare services. The current shift from the traditionally highly regulated, hierarchical and pathologising approach to THC towards a more patient-centred approach has highlighted the importance of trans patients’ satisfaction with treatment processes and results. As the still influential regulations have a negative effect on patient satisfaction, and might also keep trans individuals from seeking transition related treatment, it is crucial to investigate what trans individuals, whether patients or not, need and fear regarding transition related healthcare. Against the backdrop of mixed reactions received from the local trans community regarding the foundation of the Interdisciplinary Transgender Healthcare Centre Hamburg (ITHCCH), Germany, this study seeks to determine what trans individuals need with respect to THC in order to guarantee for high quality service provision at the ITHCCH. To this end, an online questionnaire was developed. The researchers employed a participatory approach to questionnaire development by involving a working group consisting of local trans support group representatives and (THC) specialists (*N* = 4). The sample consisted of *N* = 415 trans-identified individuals aged between 16 and 76. Most of them were based in Germany. 85.2% (*n* = 382) reported experience with transition related healthcare and 72.5% (*n* = 301) had (additional) treatments planned. Analysis revealed a need for communication and feedback opportunities. Furthermore, during the treatment process, addressing individual needs was considered crucial by participants. They agreed moderately with concerns towards THC centres. 96.5% of participants would like high decision-making power concerning treatment-associated decisions. The results demonstrate the importance of patient-centred THC that takes patients' individual needs and realities into consideration and involves patients in decision-making processes.

## Introduction

The term *transgender* (hereafter trans) refers to individuals whose gender does not (fully/constantly) match their sex assigned at birth. Trans individuals might feel they belong to the other gender (i.e., trans women, trans men), and therefore have a binary concept of gender [1]. Alternatively, they might feel they belong to both or neither genders recognised by mainstream society (i.e., male and female gender), and thus have a non-binary concept of gender (e.g., genderqueer) [1]. In order to live their gender and to feel at ease with their body, trans individuals might require a diverse set of trans healthcare (THC) services.

The study presented in this paper investigates which needs and concerns trans individuals have concerning THC offered by interdisciplinary THC centres in Germany. In doing so, the researchers sought to contribute to developing quality standards for patient-centred, interdisciplinary healthcare services for trans people. Based on the results, measures were developed to assure provision of high-quality in the Interdisciplinary Transgender Healthcare Centre Hamburg (ITHCCH). Responses from *N* = 415 individuals, the majority of which was based in Germany, were collected through an online questionnaire. The paper opens with outlining the context of THC in Germany, describes the methods used in the study, and the results obtained. It then discusses findings and implications, and presents measures of quality development implemented by the ITHCCH.

### Prevalence of trans individuals

Exact data on the prevalence of trans-identified individuals do not exist. However, the prevalence of this phenomenon has been estimated in several studies. The reported prevalence rates vary greatly, due to a highly diverse methodology: On the one hand, existing studies have used criteria that vary in exclusivity (e.g., genital surgery as the strictest criterion: 0.0043% of the Belgian population [2], legal sex and/or name change: 0.0043% of the German population [3], diagnosis of gender dysphoria, transsexualism, or gender identity disorder diagnosis: 0.0046% (results of a meta-analysis including studies from various, mostly European countries) [4], gender dysphoria diagnosis: 0.00818% (focusing on Scotland) [5], or gender ambivalence or incongruence as the widest criterion (investigated using a Dutch sample): 3.9% and 0.95%, respectively [6], or, as reported from Flanders, Belgium, 2.0% and 0.6%, respectively [7]). In their systematic review of 27 studies conducted mostly with European, American and Asian samples, Collin et al. [8] concluded a prevalence of 0.0092% for trans individuals that accessed surgical or hormonal transition related treatment, 0.0068% for trans individuals with a trans related clinical diagnosis. The prevalence of individuals self-identifying as trans was much higher (0.871%). However, this prevalence dropped to 0.355% after excluding an outlier study [9]. On the other hand, studies have based their analysis on different samples (e.g., clinical samples [4], population-based samples [6, 7]). Newer studies indicate an increasing prevalence of trans individuals [10]. Especially the numbers reported by studies using clinical samples should be seen as a minimum estimate, as not all trans individuals seek medical treatment [11].

The increasing amount of information available on the heterogeneity existing within the trans population highlights that THC is relevant to more individuals than originally expected. Simultaneously, it becomes obvious that this heterogeneous population also has heterogeneous needs that THC needs to address. Due to current diagnostic revisions (Gender Dysphoria [12], ICD-11, draft: Gender Incongruence [13]) diagnoses regarding gender identity will address a broader spectrum of people with manifold gender related issues and healthcare needs that, to this date, have not received sufficient attention from THC professionals (e.g., non-binary genders). Studies only focusing on the use of treatment services (e.g., genital reconstructive surgery, see for instance [2]) as criterion are likely to underestimate the prevalence of these phenomena, which might have negative consequences on healthcare and policy levels.

### Historic overview of trans healthcare

Historically, in Europe and North America, amongst others, trans identities have been considered from a psycho-medical, rather than a social perspective [14, 15]. This has entailed a view of the trans individual as problematic or pathological, rather than locating the problem in society and its oversimplified assumptions on sex and gender. The focus on transition related healthcare was directed to clinical diagnostics and the selection of appropriate ‘candidates’ for transition related measures. Consequently, both research and practice in the fields of medicine and psychology have sought to distinguish between “‘real’ and ‘unreal’ transsexual people” [15], obliging trans individuals to convince healthcare professionals of their trans identity to gain access to transition related healthcare.

### Transition related healthcare

#### Regulated access

Transition related treatment sought by trans individuals might include, for instance, cross-sex hormone treatment, gender-affirming surgeries, hair removal, and voice surgery. Due to the multidisciplinary requirements arising therefrom, interdisciplinary settings are most appropriate for offering transition related healthcare (i.e., THC centres [16]). Generally, trans-experienced healthcare professionals are rare and mostly located in urban areas, making access to transition related healthcare a practical and financial issue for many trans individuals.

Moreover, today’s transition related healthcare still bears characteristics derived from the aforementioned out-dated perspective on trans identities, resulting in high levels of regulation in access to, and financial reimbursement of transition related healthcare. On the one hand, healthcare professionals are provided with decision-making power and might use their gate-keeping position inherent in the existing regulations to prevent THC professionals from making wrong decisions. On the other hand, trans-identified patients might strive for self-determination on healthcare matters, but instead are obliged to conform to (or accept) a limited range of services defined solely by the providers' assumptions about what they need [15]. This system characterised by gate-keeping is likely to foster hierarchical patient–THC professional relationships with little room for trust and individuality (with the use of the term *patient* we do not imply that all trans individuals are patients, nor that there is a ‘natural’ hierarchy inherent to the term). Traditionally, this widely practised approach to transition related healthcare does not strive towards more patient-oriented healthcare processes, nor does it employ patient satisfaction as indicator for quality and success in healthcare, research, and policy. In other healthcare contexts, however, patient satisfaction is increasingly recognised as a marker for service quality [17–19] and, as a consequence, is of increasing importance when applying for funding from healthcare related government bodies [20]. This spotlight on patient satisfaction is partly due to the evolution of the patient–healthcare professional relationship towards a more equal partnership and the more active patient role associated with this development [21].

#### Differences in gate-keeping

Internationally, the degree of regulation in THC varies widely. This can be due to national regulations or attitudes of the respective healthcare professional. THC centres using the informed consent model will treat trans individuals under the sole condition of the respective patient’s ability to give informed consent [22, 23]. Conversely, in many other countries, trans individuals seeking transition related treatment might have to undergo psychotherapy and/or sterilisation in order to receive access to treatment [24, 25]. In Germany, transition related healthcare is regulated by the German Standards for the Treatment and Diagnostic assessment of Transsexuals, published in the late 1990s by a group of THC specialists [26]. Trans individuals and organisations were not included nor consulted in the process of their development, and have been harshly criticising the Standards for their pathologising perspective ever since [27]. However, it is rather the guidelines by the medical service of the Federal Network of Insurance Companies [28] that are applied by medical service task forces of insurance companies when deciding about reimbursement of transition related interventions (cf. [29]). Consequently, reimbursement of interventions for medical transition depends on several requirements, which trans activists have repeatedly linked to additional distress and impairment [27]. As both the German Standards and the Guidelines do not reflect the social, political, nor the medical needs and knowledge of today, new guidelines are currently being developed, using a participatory approach with consultations of trans support group representatives throughout Germany [29, 30]. Until the latter's publication, the Standards will require trans individuals to go through a predetermined process to attain all requirements necessary for being reimbursed for their transition related medical expenses. This includes, amongst others, a clinical assessment followed by mental health counselling and/ or psychotherapy for at least 12 months prior to commencing their hormone treatment, and, after an additional six months of counselling/ psychotherapy and hormone treatment, to be able to undergo surgeries [26]. Even though this might be handled more liberally in practice, these regulations still are highly problematic.

### Access to general healthcare

For trans individuals, accessing general healthcare is difficult on various levels. Due to the aforementioned regulations, their relationship with the healthcare system is strained and their trust is diminished [27, 31].

Moreover, the readiness to use healthcare services might be further reduced by experiences of discrimination in healthcare settings, as reported by multiple studies from various countries (e.g., [32], focusing on England, and [33], focusing on Belgium). Analysing online survey data of *N* = 6,450 trans individuals on trans discrimination in the United States, Grant et al. [1] reported that in medical settings 28% of the respondents had experienced verbal harassment, 2% had experienced physical harassment, and 19% had been refused care. The experience of discrimination or stigma varies across trans individuals, with an increased likelihood for, amongst others, members of ethnic minorities [1], individuals transitioning later in life, or individuals with low social economic status [34].

As using healthcare services can entail the unwanted disclosure of the person’s trans identity (e.g., trans men visiting the gynaecologist), reluctance to use healthcare services might be further increased. Roberts and Fantz [35] cite structural barriers to care, such as shared rooms or bathrooms during hospital stays or the mismatch between a trans individual’s appearance and their official documents. Stigma and discrimination or the fear thereof might prevent trans individuals from seeking general healthcare support [21, 36, 37].

Even if trans individuals use healthcare offers, they might have some sex-specific healthcare needs (such as cancer screenings) which might not be met because the trans body does not conform to typical male or female internal or external configuration. Against the backdrop of the high level of discrimination and stigma, trans individuals might feel reluctant to disclose their trans identity to healthcare professionals, making it impossible for their needs to be addressed [35]. Moreover, healthcare professionals often are uninformed about trans identities, THC needs, and difficulties faced by trans individuals concerning healthcare [33, 38].

### Patient satisfaction with trans healthcare services

The evidence presented thus far demonstrates a large number of problematic aspects in trans individuals’ access to transition related and general healthcare. These have negative effects on patients’ satisfaction with THC.

In the past, research in this field has mostly focused on patients’ satisfaction with surgery outcomes [39–41], their decision to transition, or their post-transition lives [40, 42, 43]. Only few studies have addressed patient satisfaction regarding the treatment process and services offered by THC centres (e.g., [20, 21, 44]). Bockting et al. [21] compared satisfaction levels of trans (*n* = 180) and cisgender (*n* = 837; i.e. individuals whose gender identity matches their sex assigned at birth) patients of a university-based sexual health clinic in the US. Wylie et al. [20] compared satisfaction levels of trans (*n* = 23) and cisgender (*n* = 31) patients of a UK-based sexual health clinic. Davies et al. [44] investigated satisfaction levels of *N* = 282 trans patients in two UK-based gender clinics.

All three studies reported high levels of patient satisfaction (78% [20], e.g., overall satisfaction 60% [44]), with no significant difference in satisfaction levels from other sexual health patients treated in the same clinic [20, 21]. However, several aspects were criticised by the participants of the three studies. As Davies et al. [44] point out, the rising number of patient referrals over the last years, coinciding with a lack of THC specialists, resulted in waiting times of six months or more for a first appointment at the UK-based clinics included in their study. Even when scheduling availability is the same as for other sexual health patients, trans participants reported a significantly lower satisfaction with availability at time 1 of Bockting et al.’s [21] longitudinal survey, indicating the great importance trans patients, often with high levels of gender dysphoria, place on prompt access to THC [45]. Moreover, the participants were dissatisfied with the THC specialists’ gate-keeping role that made it difficult to establish a trustful patient–THC professional relationship, and limited trans individuals’ autonomy in transition related decision-making [21].

All three studies used versions of the Patient Satisfaction Questionnaire [21] consisting of closed and open questions that had been adapted to the context of THC centres in the US and the UK, respectively. The instrument has yet to be validated (see [44]). The services offered and evaluated in the studies vary between clinics. The questionnaire did not address satisfaction with specific surgeries or with aspects of medical treatment, but employed rather general items on patient satisfaction. The samples consisted of patients of the respective clinics, whereas Bockting and colleagues [21] and Wylie and colleagues [20] collected data anonymously from both trans and other sexual health patients at their clinics. The anonymous questionnaire was either administered to patients before their appointments at the clinic [21], sent by post [20], or both [44].

The methodology used in these patient satisfaction surveys poses several difficulties in terms of the results’ validity. Firstly, all three studies were conducted using clinical samples of individuals that were patients in the respective THC centre. Therefore, the relationship between the participant and the researcher simultaneously is a patient–THC professional relationship and hence is likely to be characterised by dependency. The data might thus have been collected during or before the diagnostic process that determines whether or not the participant/patient will be able to receive transition related interventions. Even if the study participation was anonymous, this context might influence the replies given in the surveys. Secondly, patient samples naturally exclude individuals that have decided against undergoing transition related treatment in general or in the respective clinic. If the questionnaire is administered over a limited time period (rather than, e.g., sending it out to all patients in the clinic’s data base, or handing the survey out to all new patients), as done by Bockting et al. [21], it also statistically disadvantages individuals that seek selected interventions only, as these individuals will be less likely to be present at the clinic at the time of data collection. Thirdly, as discussed by Bockting et al. [21], patient expectations influence patient satisfaction independent of whether or not they are fulfilled [46]. Lastly, data from retrospective patient satisfaction surveys conducted by clinics are oftentimes biased by social desirability towards more positive answers [47]. The satisfaction ratings obtained in the studies discussed above might therefore only provide limited information on the actual quality of the services provided.

### Rationale

In the light of the status quo in THC and the literature discussed above, it is crucial to improve both THC access and experience. In order to align transition related healthcare services with the needs of trans individuals as much as possible, it is therefore imperative to investigate what exactly trans individuals’ needs and concerns are regarding interdisciplinary THC. Satisfaction surveys require participants to be (past) patients in THC, and therefore exclude those not seeking treatment. This approach reproduces the high hurdle for trans people to access healthcare and erases the needs and fears of those not seeking treatment. As their input is not available for consideration when modifying trans healthcare services according to results from patient satisfaction surveys, this results in a vicious circle. It was therefore necessary to recruit a more representative sample of trans people (i.e., a non-clinical sample regardless of diagnosis and transition related treatment undergone or planned) and focus on participants' needs and fears concerning transition related healthcare, rather than asking about patient satisfaction.

### The present study

In the present study, we collected data on the needs and concerns of trans individuals regarding interdisciplinary transition related healthcare. To our knowledge, there are no existent studies addressing this question in the context of THC in Germany or elsewhere. We collected data from a non-clinical online sample of trans-identified individuals. We thereby made our study inclusive of non-patients, individuals that might have decided against treatment at our THC centre or other THC services (e.g., due to low satisfaction levels), as well as prevented bias due to a participant/patient double role. Hence, our sample is likely to possess a higher level of validity than patient satisfaction studies.

The study was designed and conducted by researchers working for, or associated with, the Interdisciplinary THC Centre at the University Medical Centre Hamburg-Eppendorf. The centre is the first interdisciplinary THC centre in Germany. It was founded in 2013 and integrates eleven disciplines relevant for transition related healthcare (e.g., mental health care, endocrinology, urology, and gynaecology), offering, amongst others, mental health counselling, gender-affirming surgeries, as well as physiotherapy. The first reactions by trans individuals to the healthcare centre were positive. However, serious needs and concerns exist and have been voiced in the local trans community with regard to THC in interdisciplinary settings. In order to address both needs and concerns in the centre’s healthcare offers, the present study seeks to answer the following research question: Which needs and concerns do trans individuals have concerning THC in interdisciplinary THC centres in Germany? Even though our study focuses on the German context of THC, we expect the findings to be relevant to THC centres in other Western countries. One the one hand, trans individuals tend to form close networks for sharing information and experiences, which might lead to needs and concerns being shared and established internationally. On the other hand, needs and concerns of trans individuals regarding THC might be similar in, and the results might therefore be relevant for, countries where access to THC is regulated, and where interdisciplinary THC centres exist. As this study is exploratory in character, we did not formulate any hypotheses.

## Method

Reporting the study’s methodology and results will be done according to the Checklist for Reporting Results of Internet E-Surveys (CHERRIES) [48].

The study was approved by the ethics committee of the Chamber of Psychotherapists Hamburg (approval number 05/2015-PTK-HH). Prior to participation, participants were provided with information about the study, including approximate length of time for participation, data protection, and goals of the study, and asked to give consent (by ticking a box in the online survey). They were able to withdraw from the study during and after their participation without explanation up until two weeks after the questionnaire was closed for participation. At the beginning of the survey, participants created an individual code which kept their identity hidden, but allowed us to find and delete their answers in case they wanted to withdraw from the study.

### Questionnaire development

Following a participatory approach, the questionnaire was developed by the researchers JE, AK, and TN in co-operation with a working group consisting of two trans support group representatives and two local THC professionals (one representative, respectively, for a trans women’s support group, a trans men’s support group, one psychiatrist, and one endocrinologist). With this group line-up we sought to reflect both the THC context in the Hamburg region and the general context of THC in Germany. The authors are confident that the questionnaire developed with the working group and the issues addressed therein are relevant to THC contexts beyond Hamburg and Germany. On the one hand, access to THC is regulated in other countries outside Germany, and on the other hand, trans individuals often are well connected and might communicate on needs and concerns regarding THC on-line.

#### Participatory research

Rather than being one specific research method, participatory research represents a style of research emphasising the importance of the research context and the empowerment of traditionally disenfranchised communities [49]. Participatory approaches are increasingly employed in healthcare research, covering diverse models, projects, and approaches [50]. Reasons for participatory approaches can be of ethical, pragmatic, or political nature ([50], cf. [51, 52]). Consultation with service users at research design stage can ensure higher ethical standards in research design and higher quality of information material [53]. Furthermore, it can lead to changes in agendas and priorities and thereby increase the relevance of research questions and results [54]. Service user involvement has oftentimes become a requirement for funding bids [51]. It has been actively pursued by government bodies in the British healthcare system (i.e., NHS INVOLVE [55]) and in US healthcare research, due to a focus on practice-based evidence and discourse on patient-centred healthcare [49].

Participatory research literature reports different stages of participation. For the development of the questionnaire used in the present study, the researchers used Wright and colleagues’ stage model of participation [56]. Stages 1 and 2 represent research projects without participation (*instrumentalisation*, *instruction*). Stages 3 to 5 are pre-stages of participation (*information*, *consultation*, *inclusion*). Stages 6 to 8 represent participation in research (*participation in decision-making*, *partial decision-making authority*, *decision-making agency*). Stage 9 (*autonomy*) exceeds the participatory research approach [56]. The present study can be located in stage 5 (*inclusion*) of Wright et al.’s [56] model, which is a pre-stage of participation. If the population in question is not in control of methodology, research questions, and data analysis, the empowering effect of research on trans people is limited [57]. As participatory approaches have only rarely been applied to THC research, introducing participatory elements into research which to date has been strongly segregated from the trans population, is an important and empowering development.

Taking a participatory approach in THC research is imperative and yet a new development ([1], cf. [29, 58]). The history of THC and the traditional views still existing today call for an inclusion of trans people’s views in THC research aimed at improving THC. Moreover, the authors received input from the working group allowed the researchers to produce a questionnaire instrument that was both grounded in the diverse realities of trans individuals, trans individuals’ experience with healthcare, as well as experience from healthcare professionals working in office-based practices in contrast to the researchers’ experiences of hospital-based healthcare. Therefore, we ensure a higher validity of our results. Furthermore, by including trans individuals in the survey development, we sought to increase acceptance of, and hence participation in, the study. In doing so, we also hoped for an increased relevance of the study’s results for the trans population, which might pave the way for future co-operation. Finally, due to our participatory approach, we were able to advertise the study in local support groups, office-based practices, and socio-political initiatives (i.e., local and national charities with a focus on trans relevant issues).

#### Process of participatory survey development

The researchers were responsible for organising and chairing the working group meetings. To ensure a constant involvement of trans individuals in the process, the meetings were only held if both trans support group representatives were present. This did not apply to the healthcare professionals, who were present during one meeting each, but were able to comment on the circulated drafts and meeting protocols via email. During the first working group meeting, the research project and its objectives were presented to the working group. Based on a model tracing the transition process of trans individuals [59] and literature on patient-centred healthcare [60], the working group developed a structure for the questionnaire and compiled a list of relevant topics. Afterwards, a first draft of the questionnaire was developed by the researchers and circulated amongst the working group. The draft was discussed and revised during the second working group meeting. After the meeting, a second draft was circulated and edited based on further comments from the working group.

#### Item construction and questionnaire structure

In addition to items derived from the working group discussions, further items were taken from existing questionnaires on patient satisfaction ([61] adapted from SERVQUAL [62], PACIC 5A [63, 64]), and patient-centeredness (six items from the physician care scale of the FK-P [65, 66]). All items were adjusted to the context of THC.

The final version of the questionnaire contained, amongst others, items on socio-demographic data, trans related socio-demographic data (e.g., preferred label to describe one’s gender identity, period of time lived according to gender identity), treatment status (undergone treatment, planned treatment), needs concerning treatment process, decision-making during treatment process, concerns regarding interdisciplinary THC centres, needs concerning structural aspects of treatment process (e.g., preferred way of first contact with the THC centre), and topics deemed relevant for the THC centre’s website (please see S1 File for the items reported on in this publication). At the end of the survey, participants were able to comment on the study or other topics relevant for THC.

The questionnaire consisted of closed questions (e.g., multiple choice; statements to be rated using a 6-point Likert scale ranging from 1 *strongly disagree* to 6 *strongly agree*) and open questions. For questions regarding structural aspects and THC offers, we chose a wording that allowed participants to answer them irrespective of treatment status (*“In my view*, *it is helpful to…”*). As personal experience with THC was not used as prerequisite of participation, the participants were instructed to refer to trans-unrelated healthcare experiences when answering the questionnaire, if necessary. In order to make our questionnaire accessible to participants across Germany who might be unfamiliar with Hamburg’s THC centre, the items did not refer to this specific gender clinic. Instead, a short description of a hypothetical THC centre, bearing the Hamburg-based centre's characteristics, was described at the beginning of the questionnaire and referred to in the items.

Adaptive questioning (i.e. some questions are only displayed depending on the previous answer) was used in the final web-based version of the questionnaire to reduce the amount of questions to the necessary minimum. The number of items per page varied, and was generally kept low to minimise the amount of scrolling necessary to read all items.

#### Validation

After transferring a preliminary version of the questionnaire to online survey software, it was tested and validated by five individuals who either identified as trans individuals or had THC related knowledge to ensure that participants interpreted the items correctly. They were not familiar with the questionnaire beforehand. After further editing, one surgeon working at the THC centre checked the questionnaire for medical accuracy and relevance. A revised version of the questionnaire was sent to the working group for final approval.

### Participant inclusion and exclusion criteria

In order to be eligible for study participation, participants had to identify as trans individuals (used here as an umbrella term for, e.g., transgender, transsexual, genderqueer [1]) and be at least 16 years of age [67]. Whether participants had already accessed transition related healthcare, or had plans to do so, was not relevant concerning eligibility. To address the broadest possible spectrum of trans individuals, we sought to employ gender sensitive language.

### Recruitment process and consent procedure

To promote the study, the link, together with an invitation to the study, was emailed to a comprehensive list of trans support groups across Germany, trans activist groups, as well as trans related contacts of the researchers, and was posted in trans related groups on social network websites. Additionally, flyers were displayed in office-based practices of local THC professionals and LGBT centres. The researchers thereby sought to recruit a convenience sample that mirrored the heterogeneity of the trans population in Germany. Representation in the statistical sense was not aimed for, as information on the trans population is too limited to make such claims. Participants gave informed consent electronically by clicking ‘yes’ when asked whether they consent with their data being used for the study at the beginning of the questionnaire.

### Process of collecting data

Data collection took place over a period of two months in summer 2015, after which the survey was taken offline. Upon accessing the online questionnaire, participants first got a detailed description of the research project and its objectives. After giving informed consent to participate in the study, they were asked to generate an individual code for data protection (i.e., retaining the possibility to retrospectively withdraw from participating in the study until a date specified at the beginning of the survey). Afterwards, participants had to indicate their age (participants aged under 16 were automatically redirected to the end of the survey and hence were not able to participate). No incentives were provided for participation. Participants were only able to take part once (an IP address check was used to prevent repeated participation).

### Data analysis

#### Descriptive statistical analysis

Sample characteristics and questions regarding structural aspects of THC centres were analysed descriptively. To report participants' attitudes towards structural aspects more concisely, replies were grouped into disagreement (points 1 to 3 on the Likert scale) and agreement (points 4 to 6 on the Likert scale). Subsequently, percentages were computed.

To report the percentage of treatment completed, we computed an individual treatment status score by adding the numbers of undergone and planned treatments per participant (i.e., 100%). This enabled us to calculate the number of undergone treatments in percentage for each participant. With this score, comparing individual participants or participant groups regarding their treatment becomes possible, while taking into account the considerable variations in the number of interventions both relevant to individual participants and available for each sex.

#### Inferential statistical analysis

Inferential analyses were performed with items on treatment process, decision-making during treatment process, and fears concerning interdisciplinary THC centres. Analyses were conducted using the following five independent variables or predictors, respectively, if not indicated differently: Age, sex assigned at birth, binary/non-binary gender, and treatment status (i.e., experiences with somatic treatment versus no treatment experience or only counselling/ psychotherapy experience).

In order to reduce the number of treatment process and decision-making items, and to extract underlying factors, an exploratory factor analysis was conducted using principal component analysis and Varimax rotation. The criteria for conducting a factor analysis were met by the data (KMO = .840; Bartlett’s test *p* < .001). Missing values were excluded pair-wise. After checking for systematic missing data, no cut-off values were determined and all participants were included in the analysis. In a subsequent analysis, the sub-scale scores were modelled using a multilevel mixed-effects linear regression with the sub-scales as grouping variable within the cluster participant. The models were adjusted for the independent variables, and for all interactions between the sub-scales and these variables to show potential differences. The adjusted effects with 95%-confidence intervals were estimated and visualized. Nominal *p*-values are reported without correction for multiplicity. Two-sided *p*-values < .05 were considered significant. Multilevel mixed-effects linear regression was conducted with Stata 14 [68]. SPSS 21.0 [69] was used for all other statistical analysis.

Data regarding decision-making preferences during the treatment process were collected using three items on preference for high decision-making power for patients and three items on preference for low to none decision-making power. Additionally, two items (*I want to decide how much I am going to be involved in decisions for or against specific treatment options*; *I want the healthcare professionals and myself to share responsibility in deciding which treatment might be best for me*) could not be assigned to the subgroups and were only analysed descriptively. For data analysis we computed a score to divide participants into two subgroups (preference for high/low decision-making power in treatment process). To assign participants to the subgroups, we calculated an overall score from the sum of high decision-making power items minus the sum of the low decision-making power items. A value greater than zero indicates a preference for high decision-making power, a value lower than zero a preference for low or no decision-making power. Participants with a score of zero were excluded from the analysis, as their data did not reveal any preferences (*n* = 13). To estimate the probability of a participant belonging to one of the subgroups high or low decision-making power, respectively, a binary logistic regression was computed. Qualitative data obtained through open questions were grouped thematically and ranked according to frequency of occurrence.

## Results

### Descriptive results

The present sample consists of *N* = 415 participants (completion rate 52.4%) from across Germany (e.g., 15% from Berlin, 14% respectively from Bavaria and North Rhine-Westphalia, 10% respectively from Hamburg and Lower Saxony). 8% of participants indicated that they lived outside Germany. Main demographical data are presented in Table 1. 44.8% of the participants reported interest in interdisciplinary THC centres.

**Table 1:** Basic demographic characteristics.

Demographic Characteristics		N	%
**Sample Size**		415	
**Sex Assigned at Birth**	Assigned female	216	52.00
	Assigned male	199	48.00
**Age**^**a**^	All	38.12 ± 12.82	
	Assigned female	32.45 ± 10.76	
	Assigned male	44.28 ± 12.02	
**Country of Birth**	Germany	377	90.8
	Other European Country	28	6.7
	Non-European Country	10	2.4
**Population of Place of Residence**	< 5,000	61	14.7
	5,000–20,000	46	11.1
	20,000–100,000	61	14.7
	100,000–1,000,000	122	29.4
	> 1,000,000	120	28.9
	Cannot or would not answer the question	5	1.2
**Educational Background**	Low	32	7.7
	Medium	77	18.6
	High	288	69.4
	Other	11	2.7
	Cannot or would not answer the question	7	1.7
**Employment**	Full-time	213	51.3
	Part-time	47	11.3
	Minor employment	34	8.2
	Unemployed	73	17.6
	Retired	20	4.8
	Cannot or would not answer the question	28	6.7
**Occupational Status (multiple answers possible)**	Student	89	21.6
	In vocational training	23	5.6
	Non-skilled laborer	17	4.1
	Semi-skilled laborer	20	4.9
	Employee	195	47.3
	Public servant	14	3.4
	Self-employed	71	17.2
	Cannot or would not answer the question	25	6.1

^a^Participants assigned female at birth were significantly younger than participants assigned male, *p* < 0.001 (Mann-Whitney-U-Test).

#### Treatment status

Table 2 shows the participants’ treatment status. On average, participants assigned male at birth had undergone 4.2 treatments and had 2.7 treatments planned for the future. On average, they had completed 57.4% of their treatment. Participants assigned female at birth on average had undergone 3.7 treatments and had 2.8 treatments planned. On average, they had completed 63.9% of their treatment.

**Table 2:** Treatment status.

Assigned Females (*n* = 216)				Assigned Males (*n* = 199)			
		n (% of Cases)				n (%of Cases)	
		Undergone	Planned			Undergone	Planned
**Mental Health Counselling / Psychotherapy**	Yes	179 (92.3)	8 (6.2)	**Mental Health Counselling / Psychotherapy**	Yes	147 (94.2)	21 (14.7)
	Not sure	–	4 (3.1)		Not sure	–	2 (1.4)
**Hormone Treatment**	Yes	158 (81.4)	32 (24.6)	**Hormone Treatment**	Yes	138 (88.5)	39 (27.3)
	Not sure	–	6 (4.6)		Not sure	–	3 (2.1)
**Speech Therapy**	Yes	19 (9.8)	7 (5.4)	**Speech Therapy**	Yes	80 (51.3)	26 (18.2)
	Not sure	–	10 (7.7)		Not sure	–	9 (6.3)
				**Hair Removal**	Yes	117 (75.0)	45 (31.5)
					Not sure	–	9 (6.3)
**Mastectomy**	Yes	121 (62.4)	58 (44.6)	**Surgical Breast Augmentation**	Yes	33 (21.2)	45 (31.5)
	Not sure	–	9 (6.9)		Not sure	–	37 (25.9)
**Hysterectomy**	Yes	70 (36.1)	50 (38.5)	**Hair Restoration Surgery**	Yes	4 (2.6)	19 (13.3)
	Not sure	–	25 (19.2)		Not sure	–	30 (21.0)
**Salpingo-oophorectomy**	Yes	69 (35.6)	47 (36.2)	**Adam's Apple Surgery**	Yes	14 (9.0)	21 (14.7)
	Not sure	–	29 (22.3)		Not sure	–	30 (21.0)
**Epithesis**	Yes	15 (7.7)	26 (20.0)	**Voice Surgery**	Yes	12 (7.7)	14 (9.8)
	Not sure	–	27 (20.8)		Not sure	–	39 (27.3)
**Metoidioplasty**	Yes	23 (11.9)	29 (22.3)	**Genital Reconstruction Surgery**	Yes	70 (44.9)	81 (56.6)
	Not sure	–	44 (33.8)		Not sure	–	26 (18.2)
**Phalloplasty**	Yes	21 (10.8)	25 (19.2)	**Facial Feminisation Surgery**	Yes	13 (8.3)	36 (25.2)
	Not sure	–	46 (35.4)		Not sure	–	37 (25.9)
**Treatment of Complications**	Yes	33 (17.0)	32 (24.6)	**Treatment of Complications**	Yes	19 (12.2)	20 (14.0)
	Not sure	–	37 (28.5)		Not sure	–	40 (28.0)
**Measures to Reverse Transition**	Yes	2 (1.0)	1 (0.8)	**Measures to Reverse Transition**	Yes	1 (0.6)	1 (0.7)
	Not sure	–	9 (6.9)		Not sure	–	10 (7.0)
**Other**	Yes	7 (3.6)	3 (2.3)	**Other**	Yes	3 (1.9)	3 (2.1)
	Not sure	–	3 (2.3)		Not sure	–	6 (4.2)

#### Structural aspects of trans healthcare

In the following, participants’ needs concerning structural aspects of interdisciplinary THC centres, such as contact options, will be presented.

#### Initial contact with THC centre

Email was reported as preferred option for initial contact with a THC centre by 36.4% of participants, followed by open consultation hours (18.1%), and phone calls (13.7%). Using the comment section available for this question, a high number of participants underlined that a THC centre should offer more than one contact option, including at least one anonymous option, to cater to diverse patient needs. The same applied for *regular contact person* and *post-treatment feedback*.

#### Regular contact person

Most participants (91.8%) preferred a regular contact person for feedback and critique during the treatment process. Preferred contact options were email (27.7%) and face-to-face conversation (27.5%).

#### Post-treatment feedback

The vast majority of participants (96.5%) would like the possibility to give feedback upon completion of their treatment. Preferred options were an anonymous online form (26.8%), a face-to-face conversation with their psychotherapist (25.8%), and email (24.6%).

#### Peer support

The majority of participants (94.7%) were in favour of THC centres establishing a peer-support programme with former patients supporting current patients. The most relevant aspects for support were better coping with treatment-associated stress in everyday life and the exchange of experiences. *N* = 22 participants (5.3%) opposed peer support. Reasons for refusal of peer support given in the optional open answer section provided for this question were fear of dependence (*n* = 7) and already existing peer networks within the trans community (*n* = 8).

#### Co-operation between THC centre and support groups

38.3% of the participants were in favour of support groups co-operating with THC centres in a consulting capacity. 35.4% preferred a regular exchange between a THC centre and local support groups, with the latter remaining independent. 5.5% opposed any co-operation between THC centres and support groups. Qualitative replies revealed that fear of lacking independence from THC centres were the major reason for this opposition.

#### Research

Nearly all participants (93.5%) supported research in the field of THC. 88.5% also reported that they would agree to participate in trans related research conducted at THC centres. *N* = 46 participants (11.5%) indicated they would not like to participate in future research. Reasons for not participating in future research were, among others, ethical concerns regarding research methods (*n* = 14), such as the assumption that research results could be used to back pathologising views on trans individuals, and the misuse of the data for economical purposes by THC centres (*n* = 10). 95.8% of the participants want to get informed on important results regarding trans related research.

#### Healthcare offers

In the following, the participants’ preferences regarding healthcare offers (e.g., follow-up care) are presented.

#### Surgical follow-up care

Most participants (93.0%) would be interested in offers of follow-up care by the responsible surgeon. Thereof, 63.3% would like to make appointments if required, 29.4% preferred to make several fixed appointments right after surgery. For aspects of surgical follow-up care, multiple answers were possible. 84.6% of the participants stated the wish to receive assistance and support in dealing with surgery results. 55.2% reported the wish for post-operative physiotherapy. 45.5% supported post-operative mental health counselling. Other relevant aspects reported in open questions were pain management and corrective surgery.

#### Rehabilitation

80.3% of the participants reported post-operative day-patient or inpatient rehabilitation measures as helpful. Thereof, 76.1% were in favour of a co-operation between THC centres and external rehabilitation clinics.

#### General healthcare

The vast majority (93.1%) were interested in trans-specific general healthcare offered by THC centres. The following aspects were valued as important: support regarding contact with health insurance companies (85.3%), cancer screening (67.7%), measurement of bone density (51.3%), and fertility options (49.4%). Qualitative data yielded a reported need for counselling regarding sexually transmitted diseases, gynaecological and/or urological support, as well as post-operative treatment after genital reconstructive surgery. Analysis of open answers revealed that the main reasons for treatment in an interdisciplinary THC centre are the provision of a ‘safe space’ (*n =* 228), professional competence (*n =* 132), holistic treatment (*n =* 35), and organizational aspects (e.g., short distances between different healthcare professionals; *n =* 24). Reasons against treatment in an interdisciplinary THC centre were the segregation of trans people from general healthcare (*n* = 6), limitations of the free choice of THC professionals by patients (*n* = 3), and a poor accessibility of the centre (*n* = 3).

#### Mental health care

Nearly two-third (74.9%) of participants considered mental health counselling as helpful during their transition. 58.1% of those participants also considered mental health counselling in an integrated setting (i.e., a THC centre) helpful. Regarding the frequency of appointments, 38.1% preferred sessions every second or third week, and 32.5% every four to eight weeks. Only 17.7% preferred weekly appointments. 85.6% considered the option of telemedical treatment as helpful. The following telemedical options were considered best: Email (34.3%), phone call (32.0%), and video-supported phone call (16.6%). Participants also considered mental health counselling in the ward, shortly before (56.4%) and after surgery (56.3%), helpful. The option to use psychotherapy to deal with trans-unrelated issues (e.g., depression) was considered helpful by 88.0% of participants.

#### Fears concerning interdisciplinary THC centres

On average, participants moderately agreed with the fears associated with THC in an interdisciplinary setting presented in the items (for detailed results see Table 3). Qualitative answers yielded the following additional themes: having to travel long distances for treatment in a THC centre, long waiting times, fear of THC centres institutionalizing pathologising perspectives on trans identities, free choice of THC professionals being limited, lack of consideration of individual needs concerning treatment, as well as THC centres having predominantly economical motivations at heart, rather than their patients’ interests and needs.

**Table 3:** Fears associated with THC centres.

Items	n	Mean ± SD
I am afraid that interdisciplinary THC centres will…		
monopolize THC.	411	3.85 ±1.45
prevent me from being able to choose where I go for trans related treatment.	410	3.96 ± 1.54
result in THC professionals expecting me to undergo a certain number of trans related treatments.	411	3.24 ± 1.65
result in my not being able to influence what type of treatment I will get.	410	3.66 ± 1.55
lead to my being confronted with ever-changing THC professionals during one single treatment.	412	3.65 ± 1.44

### Inferential results

#### Requirements towards THC

In order to reduce the amount of items, a factor analysis was conducted. It revealed five factors (i.e., sub-scales) explaining 51.8% of total variance. Two sub-scales lacked high factor loadings and could not be interpreted. Therefore, the analysis was recomputed with a predetermined number of three sub-scales, thus retaining three factors from the first factor analysis, explaining 41.8% of total variance. Bearing in mind that the items used for the analysis exhibited ceiling effects, this percentage is acceptable. The three remaining sub-scales were named *Communication and Social Support*, *Individual Care* and *High-Quality THC Professionals* (see Table 4), which were subsequently used as dependent variables in a linear regression analysis.

**Table 4:** Factor structure of treatment process questions.

Sub-Scale (Factor)	Example Item	Mean ± SD
**Communication and Social Support**	I must be asked how work, family, or my social situation might influence my treatment.	4.43 ± 0.79
**Individual Care**	The order of treatments must be tailored to my individual needs.	5.38 ± 0.52
**High-Quality THC Professionals**	The healthcare professionals must provide me with easy-to-understand answers to my questions.	5.24 ± 0.54

The sample size for the multilevel mixed-effects linear regression analysis was *n* = 412 with an average of three observations per participant. Three participants had not given any information on whether or not they had any treatment experience, could not be categorized properly and were excluded. With regard to inferential analysis comparing subgroups, the analysis showed a significant effect for sex assigned at birth on the *Individual Care* sub-scale (see Table 5). Participants assigned female at birth reported significantly higher values than those assigned male at birth (*b* = -0.17, *p* = 0.015). Moreover, a significant effect for transition related somatic treatment experience was found for the sub-scale *Communication and Social Support*. Participants who already had undergone somatic treatment reported significantly higher values than participants not having undergone somatic treatment (*b* = -0.36, *p* > 0.001).

**Table 5:** Results of subgroup analyses of factors.

Independent Variables (*b*, *p*-value)	Factor (Dependent Variable)		
	Communication and Social Support	Individual Care	High-Quality THC Professionals
**Age**	n.s.	n.s.	n.s.
**Sex Assigned at Birth**	n.s.	*b* = -0.17, *p* = 0.015 (male: 5.43 ± 0.43; female: 5.33 ± 0.59)	n.s.
**Binary/Non-Binary Gender**	n.s.	n.s.	n.s.
**Treatment Experience**	n.s.		n.s.
**Treatment Phase**	*b* = -0.36, *p* > 0.001 (no somatic treatment: 4.37 ± 0.80; somatic treatment: 4.63 ± 0.70)	n.s.	n.s.

#### Decision-making during the treatment progress

We analysed the data of *n =* 402 participants concerning their preferences of high/low decision-making power during their treatment. The score calculated from the participation items revealed that *n =* 388 (96.5%) of the participants want to be in charge of decisions regarding their treatment. Only *n =* 15 participants (3.5%) want to (partly) relinquish their power to make decisions regarding their treatment to healthcare professionals.

A binary logistic regression analysing possible predictors of group membership (high/low decision-making power during treatment) did not yield statistically significant results for the independent variables tested (e.g., sex assigned at birth: Wald- χ^2^ = 1.44, *p* = 0.23; age: Wald- χ^2^ = 0.65; *p* = 0.42).

The two additional items on decision-making obtained medium to high approval (*I want to decide how much I am going to be involved in decisions for or against specific treatment options*: mean value = 5.53; SD = 0.76; *I want the healthcare professionals and myself to share responsibility in deciding which treatment might be best for me*: mean value = 4.33; SD = 1.57).

## Discussion

With the present online study investigating trans individuals’ needs and concerns regarding THC in interdisciplinary THC centres, we sought to support the development of quality standards for patient-centred, interdisciplinary THC. Using a participatory approach, we were able to incorporate the perspectives of trans support group representatives and THC professionals into the questionnaire design. In the following, we will highlight and discuss key results, address implications for THC, including measures developed to guarantee quality at ITHCCH, discuss limitations of the study, and suggest areas of further research. When discussing our results, we will first concentrate on characteristics of our sample and contrast it to samples used for other THC studies, such as the relatively high number of non-binary identified participants. We will then focus on participants’ needs with regards to THC, such as easy access to THC, having one’s individuality and preferred level of involvement in decision-making respected during treatment. Furthermore, we will then discuss participants’ concerns, which were less pronounced than expected.

### Sample characteristics

We succeeded in recruiting more than twice the number of participants originally expected, which might be partly due to a higher acceptance of the study within the trans-identified population based on our participatory approach. However, our sample is not representative for the trans population in Germany (see *Limitations*).

Almost half the sample approves of different THC treatments being offered together in THC centres. This makes our results extremely relevant for other THC centres seeking to provide high-quality, patient-centred services.

#### Non-binary genders

Our analysis revealed a very high percentage of participants identifying (at least partly) as non-binary. Non-binary genders have been, and often still are, unknown to, or misunderstood by THC professionals, and might serve as a reason to refuse access to transition related treatment. Consequently, non-binary individuals seeking treatment might feel discouraged from actively exploring their non-binary gender during, for example, clinical assessment. Moreover, they might keep their non-binary concept of their gender hidden as passing as a binary trans person might increase their chances of accessing treatment (cf. [70, 71]). The current or soon to be published versions of the medical classification lists DSM [12] and ICD [72] do not adhere to a binary gender perspective, which will most probably make transition related interventions more accessible for non-binary individuals. Unlike most studies on THC, the present study used a non-clinical sample (see *Methods*). As our results were thus obtained outside diagnostics and clinical contexts, the high number of non-binary individuals might forecast future clinical realities and samples resulting from DSM and ICD revisions (see also prevalence reported by [6, 7]). Moreover, the distribution of non-binary genders across male/female sex assigned at birth was uneven, with most non-binary participants having been assigned female at birth. This corresponds with other studies [73–75], as well as with clinical experiences from THC professionals at our centre.

#### Age

The age distribution differs from those reported in clinical studies [41, 76]. The authors expect that the present non-clinical sample is closer to the actual age distribution of trans individuals in society. This difference from clinical samples is due to the fact that data from clinical samples usually are collected when trans individuals first present at the respective clinic for transition related interventions. Our sample, however, includes participants at all stages of social and medical transition.

#### Treatment status

The sample included participants at all stages of medical transition. Our results on needs and concerns of trans individuals in THC settings therefore possess a high validity and relevance for quality development in THC settings. Our results furthermore reveal that the number of treatments necessary for trans individuals to consider their medical transition as complete is highly individual (e.g., some might consider only hormone treatment necessary and therefore would see their transition as complete when starting to take hormones, whereas others might only consider their transition complete after both hormonal and surgical treatment). Our findings therefore demonstrate that the concept of a ‘full’ treatment is not appropriate (e.g., both hormone treatment and genital surgeries). Rather, it seems appropriate to consider several options equally relevant either in single use (e.g., penile epithesis *vs*. phalloplasty) or in sequential use (e.g., starting to live with a packer, then trying to stay with a metoidioplasty, and ending with a phalloplasty years after first intake at the THC centre). THC professionals therefore should more easily accept singular intervention requests from patients.

### Needs concerning THC

#### Structural aspects of THC

Our results revealed a need for easy access to THC, which corroborates findings from Bockting et al. [21] and Davies et al. [44]. According to Bockting et al. [21], the strong need for easy access to THC reported by trans individuals might be due to their marked gender dysphoria, making long waiting times for a first appointment hard to bear. As our data revealed, this could be achieved by taking the diverse needs of trans individuals seeking treatment into consideration (e.g., providing a range of contact options in order to also cater to trans individuals who might be uncomfortable with talking on the phone, e.g., due to their voice range not matching their gender identity).

Our results show that, in order to consider individual needs and concerns during the treatment, and to be able to continuously improve its services, THC centres should assign patients a regular contact person during their treatment, and provide the possibility for (one-to-one) feedback at the end of treatment. The high importance attached to feedback and communication between patients and THC professionals by our sample again highlights a great need to attend to patients’ individual needs and concerns during treatment.

Furthermore, the strong approval of peer support programmes to support patients during treatment apparent from our data demonstrates the need for information and support. Additionally, involving former patients in the trans patient care would increase the exchange between trans individuals and THC professionals and, therefore, might be another step towards a more transparent, participatory, and high-quality THC. Here, also a co-operation with support groups can help to assure easy access to and high quality of THC. Simultaneously, comments from our participants opposing any contact between support groups and THC centres again show a high level of distrust in THC centres.

Even though most participants in our study recognise the importance of research and would agree to participate in studies during their treatment at a THC centre, many trans individuals seem to distrust trans-focused research. It therefore is crucial to produce high-quality research, which is ethically sound and provides support for a more liberal approach to THC, rather than cementing a pathologising perspective. Participatory approaches to research, as employed in the present study, can further increase trust by (partially) overturning the participant–researcher divide.

#### Healthcare offers in THC centres

Our results reveal a high interest in support according to individual needs (such as follow-up care or rehabilitation). Reasons for interest in general healthcare offers reflect the participants' hope of being treated in a non-discriminatory and trans-experienced atmosphere (see *Theoretical Background*). As numerous participants in our study opposed trans-specific offers in general healthcare, often based on fears of further exclusion from society, general healthcare should be offered both by THC centres and office-based practices (e.g., such as GP or gynaecologist surgeries). In order to realise these widespread healthcare offers for trans individuals, trans related issues have to be integrated more strongly into university curricula for medical students [77, 78]. Additionally, THC centres should offer support and training to other professionals working in the context of THC.

Even though mandatory clinical assessment and mental health care during the treatment process (see Standards, [26]) is strongly criticised, the majority of our sample considers options for mental health care (counselling, psychotherapy) helpful. Radix and Deutsch [79] reported a high demand for both when offered as optional service in THC centres working with the informed consent model. This highlights again that non-mandatory trans-informed mental health care is important to many trans individuals during treatment. The problematic issue therefore is the patient–THC professional hierarchy, making the establishment of a trusting relationship difficult (cf. [29, 80]). Additionally, the fact that in Germany and many other countries (e.g., the Netherlands, Belgium, the UK, Sweden) counselling with a mental health professional has to be attended for a certain amount of time before hormone treatment, thus prolonging the waiting time for medical transition, is considered highly problematic by our sample.

Our results on frequency and number of counselling sessions preferred by the participants is comparable to results reported by Bockting et al. [21].

#### Decision-making during the treatment progress

Our results showed that the vast majority of participants prefer to have high decision-making power regarding the treatment process. This mirrors the concerns voiced by trans representatives against the German Standards [26] which are currently in the process of a fundamental revision. Furthermore, it corroborates our findings concerning individual care and concerns (see below). The individuals in our sample preferring high decision-making power and those preferring low or no decision-making power could not be distinguished by socio-demographic characteristics. It is therefore not possible to infer decision-making preferences from, for instance, an individual's age or assigned sex. Modern healthcare ethics demand patient autonomy with regards to treatment. These results therefore do not recommend subjecting patients to the will of THC professionals concerning decisions. They rather suggest that a small group of participants prefer THC professionals to take responsibility and a pro-active stance concerning decisions. Thus, decision-making preferences should explicitly be discussed at the beginning of treatment. However, to support THC specialists in implementing a collaborative model of decision making, structural changes (e.g., in medical education, trans related policy) should be realised [80].

In order to facilitate the implementation of a collaborative model of decision making, THC specialists should have access to trans related information and training on collaborative decision making in their education.

#### Requirements towards THC

Our results revealed that having one’s individual needs recognised and catered to (in the following referred to as ‘individual care’) is highly important for trans patients during their treatment. This might include the possibility to address individual needs concerning types of interventions, organisational aspects (e.g., timing of surgeries), or considering the patient's individual social situation during the medical transition. The attention accorded to the aspect of individual care might be a reaction to the highly regulated access to treatment, which does not put patient satisfaction first and leads to fear and distrust (see *Theoretical Background*). Individual care is a main feature of patient-centred healthcare. Our results therefore again underline the need for change in THC.

Female participants valued individual care during treatment significantly more than male participants. This might be due to the once popular interpretation of female trans identities as a sexuality related issue (e.g., autogynephilia), rather than an identity related issue (as usually done with male trans individuals, cf. [81]).

Furthermore, participants who had already undergone transition related interventions rated communication and social support as more important than individuals without transition related treatment experience. This might be due to the former's first-hand experience of the treatment process. The latter might focus on surgery outcomes and relief from gender dysphoria as most important aspect during treatment, which might lead to underestimating the importance of communication and social support. The fact that individuals with somatic treatment experience emphasise these aspects of the treatment further demonstrates that they should be considered systematically by THC centres. Consultations with treatment experienced trans support group representatives, or forming an advisory board with treatment experienced trans individuals, might be helpful to include appropriate measures in the treatment process.

### Concerns regarding interdisciplinary THC centres

In the section on concerns regarding THC in interdisciplinary THC centres, our questionnaire addressed fear of potential monopolisation and limitation of patients’ choice of THC professionals. Furthermore, it focused on expectations to undergo a specific number of interventions, of not being able to decide on the type of interventions, and of being confronted with ever changing health care professionals during treatment. The concerns regarding these aspects were less pronounced than expected. However, participants voiced their concerns in their answers to many open questions across the questionnaire, sometimes as reason for not undergoing any treatment, or deciding against undergoing treatment in a THC centre. As individual care was considered highly important by our sample (see above), and distrust against healthcare services is widespread across the trans population [82], these fears have to be a strong concern for THC centres and THC policy makers. We expect that moving away from a highly regulated THC system towards a more transparent and individual model of care will reduce the level of concerns and distrust towards THC within the trans population. Moreover, it is crucial to work towards establishing THC in rural areas to make access to high-quality THC easier for more trans individuals ([27], see also [44]).

### Implications of the results

The results from our study aim to serve as a basis for quality development for interdisciplinary THC centres offering patient-centred high-quality services. Next to findings from existing patient satisfaction studies, input from trans individuals as well as support and advocacy groups, experience from THC professionals, they can help to develop measures for quality development.

#### Measures for quality development

The interdisciplinary THC centre in Hamburg is working at implementing several changes, based on the results from the present study. As easier access was deemed important by our participants, the centre now offers several options for initial contact (phone call, email, online form) instead of just one. Due to participants’ indicating that support with surgery results would be considered helpful, the services offered now include physiotherapy (mostly aimed at trans women for pre- and post-surgical pelvic floor training), and physiotherapists working at the University Medical Centre have been informed about the THC centre in order to further integrate physiotherapy into the THC services. Colleagues from most disciplines belonging to the THC centre have been informed about the study results and were able to discuss their experiences. One topic explicitly discussed was the treatment of non-binary trans patients. Moreover, the mental health care professionals’ knowledge on non-binary genders has been deepened further in a workshop.

For the future, the THC centre seeks to further improve its partnership with the local trans population. One option would be establishing an advisory board with representatives from local support groups (see [21, 83]). An additional measure to increase transparency and, as a consequence, reduce distrust and misinformation, is the launch of a THC centre website offering information on transition related services, THC professionals, treatment process, as well as other support or THC available locally.

In order to measure the impact of the actions taken, a longitudinal patient satisfaction survey could be implemented.

### Limitations

#### Participatory questionnaire development

Employing a participatory approach to THC research represents an important development, which can lead to the recognition of diverse identities and realities [75], and can thus empower both those trans individuals involved in the questionnaire development and those participating in the study.

However, participatory approaches to research have been criticised of “romanticizing ‘Community’ [and] disguising the powerful […] [by] essentializing the word community as a homogeneous entity where people have egalitarian interests” [84]. Hierarchy and conflicting interests existent within communities therefore are ignored by researchers external to the community, which in turn enforces an inadequate perspective onto the community and further disempowers the powerless. As the trans population is extremely diverse and controversial in terms of gender concepts, (non-)binary genders, and perspectives on THC, not to mention socio-demographic and economic differences, construing the trans population as a homogeneous community appears to be a contortion of reality.

No representatives of non-binary trans people were included in the working group. Likewise, concerns of trans people not participating in support groups or living in rural areas were not represented. The trans-identified members of our working group both had a white, rather well-educated and, to our knowledge, economically stable background. However, as representatives and long-time facilitators of support groups accommodating a large and diverse group of trans people, the working group members were deemed able to report on other trans people’s needs and concerns. We are therefore confident that the diversity of the local trans population was sufficiently addressed in the questionnaire.

#### Participant recruitment and sample

As the researchers heavily relied on trans support groups for participant recruitment, fully-closeted trans individuals (i.e., individuals who have not revealed to others that they identify as trans) and trans individuals isolated from trans-supportive structures might be under-represented in our sample. However, many trans or trans-questioning individuals (i.e., individuals who are not sure whether they identify as trans) use the internet for advice and support [85] and consult trans-specific forums or websites. As our link was posted in trans related groups on social media websites and was furthermore posted on other trans related websites and in online forums for trans people, we are confident that also these rather isolated parts of the trans population are represented in the survey. The fact that not all participants reported contact to support groups or peers, or treatment experience, further corroborates this conclusion. Moreover, parts of the trans population that might be disadvantaged and hard to reach by an online survey (e.g., those living in rural areas, having lower levels of education, or being unemployed) were represented in the sample (see Table 1) with percentages comparable to other trans samples used in research (US sample: high education level: 55%, unemployed: 14%, rural place of residence: N/A [1], European sample: high education level: 49%, unemployed: 14%, rural place of residence: 14% [82], our sample: high education level: 69.4%, unemployed: 17.6%, rural place of residence: 14.7%. The comparison shows that our sample contains more highly educated individuals, whereas numbers of unemployment and living in rural areas are similar.). However, these comparisons should be considered with caution as the samples are from other countries. Due to reporting of low educational achievements being inconsistent across the studies cited here, we refer to the percentage of participants with high educational achievements instead.

Even though the researchers sought to make the recruitment more inclusive, individuals experiencing barriers to participating in online questionnaires (due to, e.g., language barriers, low education, illiteracy, lack of computer literacy) were excluded (this might include migrant trans sex workers). The high percentage of participants holding a university degree and being employed might be an indicator for the data being biased in favour of these groups. As these groups possibly excluded from the survey might be amongst those experiencing problems in accessing THC services, improvement of future sampling strategies is needed.

Furthermore, the use of inclusive language (i.e., language sensitive to diversity and non-binary genders) in the invitation to participate and in the questionnaire itself also led to the exclusion of transsexual-identified individuals. As reported in the comments or mentioned during face-to-face conversations with the researchers, some transsexuals felt excluded by the terms used. Our efforts to be as inclusive as possible might have partly failed, leading to a possible overrepresentation of trans individuals with a social constructivist perspective on gender in general and trans identities in particular.

### Future research

Future research should aim for a higher level of participation (see [56]). Participatory designs are highly recommended for research on healthcare quality in order to ensure validity of results and the development of measures to improve the quality of healthcare. Especially in THC, individuality, flexibility, communication and high quality interventions are paramount.

In order to trace developments in trans identities and treatment processes over time, as well as to analyse causal relations, future studies should be conducted longitudinally with a fixed set of participants.

### Conclusion

This study represents a new and positive development on different levels. Participatory research so far has only rarely been employed in the context of THC. Next to few other studies [1, 58], the present study has shown that against the backdrop of the difficult history of THC, ongoing discrimination, and trans individuals’ minority status in society, a participatory approach to THC using a non-clinical sample is important. Furthermore, the results paint a detailed picture of trans individuals’ varied needs and concerns regarding THC in Germany, such as the importance of an individual and interdisciplinary approach to care, communication, and the possibility for feedback and to participation in decision-making during treatment. Furthermore, the results showed that participants’ concerns were less pronounced than expected. These insights should be used to improve existing THC offers. Locally at the ITHCCH, the study has led to positive changes to assure high-quality treatment. By developing practical measures on the basis of our results and by successfully implementing them, THC centres may help pave the way to a high-quality, patient-centred THC that caters to trans individuals' individual needs.

## Supporting information

S1 File Interdisciplinary trans healthcare survey. List of items used to collect the data reported in this publication.
(DOCX)
