# Supplementary material for: Needs and concerns of transgender individuals regarding interdisciplinary transgender healthcare: A non-clinical online survey
Source: PLoS One. 2017 Aug 28;12(8):e0183014. doi: 10.1371/journal.pone.0183014 (PMC5573291; doi:10.1371/journal.pone.0183014)
Supplement: S1 File — List of items used to collect the data reported in this publication. (DOCX) [file pone.0183014.s001.docx]

**Interdisciplinary Trans Healthcare Survey**

**Personal information and socio-demographic data**

1. **How old are you?** OPEN ANSWER
2. **What is your country of birth?**
   - Germany
   - European country except Germany
   - Non-European country
3. **How many inhabitants does your place of residence have approximately?**
   - Less than 5,000 (village)
   - Between 5,000 and 20,000 (small town)
   - Between 20,000 and 100,000 (town)
   - Between 100,000 and 1,000,000 (city)
   - More than 1,000,000 (major city)
   - I don’t know
4. **What is your level of education?**
   - Dropped out of school
   - Certificate of Secondary Education (9 years of education)
   - General Certificate of Secondary Education (11 years of education)
   - Entry qualification for advanced technical college/ university of applied science
   - A-Levels, general entry qualification for university
   - Other qualification, please specify: OPEN ANSWER
   - University degree (university, university of applied science)
   - I don’t know
5. **What is your current level of employment?**
   - Full-time
   - Part-time
   - Minor employment
   - Unemployed
   - Retired
   - I don’t know
6. **What is your current/last occupational status?**
   - Student
   - In vocational training
   - Non-skilled labourer
   - Semi-skilled labourer
   - Employee
   - Public servant
   - Self-employed
   - I don’t know
7. **Which term(s) describe(s) your gender identity best? [MULTIPLE ANSWERS POSSIBLE]**
   - Woman
   - Man
   - Trans woman
   - Trans man
   - Trans*
   - Trans
   - Transgender
   - Trans identified
   - Transsexual
   - Bigender
   - Genderfluid
   - Genderqueer
   - In between
   - Inter*
   - Inter
   - Agender
   - I can’t or don’t want to label my identity
   - Other, please specify: OPEN ANSWER
8. **What is your gender assigned at birth?**
   - Female
   - Male
9. **Have you accessed treatment (psychotherapeutic, clinical) in the past to alter your sex characteristics?**
   1. **Filter:** YES. Which trans related treatment offers have you used/ are you using? Please specify for each treatment type.

For male-to-female individuals:

| **Treatment type** | **Yes, accessed** | **No, not accessed** | **For me personally, it would have been helpful to access this treatment in the context of a trans healthcare centre.** | **Reasons** |
| --- | --- | --- | --- | --- |
| **Mental Health Counselling / Psychotherapy** |  |  |  |  |
| **Hormone Treatment** |  |  |  |  |
| **Hair removal** |  |  |  |  |
| **Speech therapy** |  |  |  |  |
| **Surgical breast augmentation** |  |  |  |  |
| **Genital reconstruction surgery** |  |  |  |  |
| **Adam’s apple surgery** |  |  |  |  |
| **Voice surgery** |  |  |  |  |
| **Facial feminisation surgery** |  |  |  |  |
| **Hair restoration surgery** |  |  |  |  |
| **Treatment of complications** |  |  |  |  |
| **Measures to reverse transition** |  |  |  |  |
| **Other, please specify** |  |  |  |  |

- 1. **Filter:** No. Why not?
- OPEN ANSWER

For female-to-male trans individuals:

| **Treatment type** | **Yes, accessed** | **No, not accessed** | **For me personally, it would have been helpful to access this treatment in the context of a trans healthcare centre.** | **Reasons** |
| --- | --- | --- | --- | --- |
| **Mental Health Counselling / Psychotherapy** |  |  |  |  |
| **Hormone Treatment** |  |  |  |  |
| **Speech therapy** |  |  |  |  |
| **Chest surgery (Mastectomy)** |  |  |  |  |
| **Removal of uterus (Hysterectomy)** |  |  |  |  |
| **Removal of ovaries (salpingo-oophorectomy)** |  |  |  |  |
| **Epithesis** |  |  |  |  |
| **Clit-Pen (Metoidioplasty)** |  |  |  |  |
| **Penile reconstruction (Phalloplasty)** |  |  |  |  |
| **Treatment of complications** |  |  |  |  |
| **Measures to reverse transition** |  |  |  |  |
| **Other, please specify** |  |  |  |  |

1. **Filter:** NO. Why not?

- OPEN ANSWER

1. **Are you planning to access (additional) treatment (psychotherapeutic, clinical) in the future in order to alter your primary sex characteristics?**
2. **Filter:** YES. Which transition related treatment measures would you like to access?

For male-to-female individuals:

| **Treatment type** | **Yes, I want to access this** | **No, I don’t want to access this** | **I don’t know** | **For me personally, it would be helpful to access this treatment in the context of a trans healthcare centre.** | **Reasons** |
| --- | --- | --- | --- | --- | --- |
| **Mental Health Counselling / Psychotherapy** |  |  |  |  |  |
| **Hormone Treatment** |  |  |  |  |  |
| **Hair removal through epilation** |  |  |  |  |  |
| **Speech therapy** |  |  |  |  |  |
| **Surgical breast augmentation** |  |  |  |  |  |
| **Genital reconstruction surgery** |  |  |  |  |  |
| **Adam’s apple surgery** |  |  |  |  |  |
| **Voice surgery** |  |  |  |  |  |
| **Facial feminisation surgery** |  |  |  |  |  |
| **Hair restoration surgery** |  |  |  |  |  |
| **Treatment of complications** |  |  |  |  |  |
| **Measures to reverse transition** |  |  |  |  |  |
| **Other, please specify** |  |  |  |  |  |

1. **Filter:** No. Why not?

- OPEN ANSWER

For female-to-male trans individuals:

| **Treatment type** | **Yes, I want to access this** | **No, I don’t want to access this** | **I don’t know** | **For me personally, it would be helpful to access this treatment in the context of a trans healthcare centre.** | **Reasons** |
| --- | --- | --- | --- | --- | --- |
| **Mental Health Counselling / Psychotherapy** |  |  |  |  |  |
| **Hormone Treatment** |  |  |  |  |  |
| **Speech therapy** |  |  |  |  |  |
| **Chest surgery (Mastectomy)** |  |  |  |  |  |
| **Removal of uterus (Hysterectomy)** |  |  |  |  |  |
| **Removal of ovaries (salpingo-oophorectomy)** |  |  |  |  |  |
| **Epithesis** |  |  |  |  |  |
| **Clit-Pen (Metoidioplasty)** |  |  |  |  |  |
| **Penile reconstruction (Phalloplasty)** |  |  |  |  |  |
| **Treatment of complications** |  |  |  |  |  |
| **Measures to reverse transition** |  |  |  |  |  |
| **Other, please specify** |  |  |  |  |  |

1. **Filter:** No. Why not?

- OPEN ANSWER

**Questions regarding trans healthcare**

Initial contact with trans healthcare centre

1. **In my view, it is helpful to be offered the following option for initial contact to an integrative, interdisciplinary healthcare offers [PLEASE SELECT ONE OPTION].**We understand that you might prefer more than one way of establishing initial contact. However, we would like to ask you to indicate which option you prefer most.
   1. Registration via phone call
   2. Online form
   3. Email
   4. Advice via phone call (Open office hours)
   5. Face-to-face advice without appointment (Walk-in clinic)
   6. Online open consultation hour, e.g. online chat
   7. Other, please specify: OPEN ANSWER

Treatment process

| On the following six pages you will find statements concerning the treatment process in the context of trans healthcare.  We would like to know which of the listed aspects **absolutely** **must** be part of good trans healthcare, which aspects are **less imperative**, and which **do not have to** be part of good trans healthcare.  It is possible that you might find most aspects important. We would still ask you to rate your answers, so that the study produces meaningful results. Please use the rating scale on the right hand side to indicate whether you agree or disagree with the respective statement.  **Example:** If you think that the respective statement describes an aspect that absolutely must be part of good trans healthcare, please select “I strongly agree” on the rating scale. If you however find that the statement describes an aspect which is less imperative for good trans healthcare, please select a rating point closer to the middle of the scale, etc. | **Likert scale 1-6: *I strongly disagree* to *I strongly agree*** | ***N/A*** |
| --- | --- | --- |
| “I **must** be asked about my goals with regards to improving my life as trans individual.” |  |  |
| “The involved healthcare professionals **must** provide me with information tailored to my individual needs.” |  |  |
| “During the treatment planning phase I **must** be asked about my individual expectations.” |  |  |
| “I **must** be able to discuss my individual expectations regarding surgical treatment with the surgeons.” |  |  |
| “I **must** be encouraged to attend or visit (support) groups or establishments for trans individuals.” |  |  |
| “I **must** be offered support, guidance and advice with regards to coming out as trans.” |  |  |
| “There **must** be counselling offers for my next of kin/ family on trans related issues.” |  |  |
| “I **must** be asked how work, family, or my social situation might influence my treatment.” |  |  |
| “I **must** be offered the opportunity to access social counselling services (e.g., regarding work, housing).” |  |  |
| “It **must** be made sure that my habits and way of living are taken into account when my treatment is planned.” |  |  |
| “The order of treatments **must** be tailored to my individual needs.” |  |  |
| “It **must** be possible to have breaks in between the single treatments if I need it.” |  |  |
| “I **must** be given the opportunity to regularly reflect on the treatment.” |  |  |
| “I **must** be able to discuss insecurities with regards to decisions for or against single treatments.” |  |  |
| “My individual needs **must** be taken into account at all times.” |  |  |
| “The time schedule agreed with me **must** be adhered to.” |  |  |
| “I **must** be able to trust the healthcare professionals.” |  |  |
| “Healthcare professionals **must** be competent enough to adequately answer my questions.” |  |  |
| “Healthcare professionals **must** provide me with easy-to-understand answers to my questions.” |  |  |
| “Healthcare professionals **must** be empathetic and understanding towards me.” |  |  |
| “I **must** be provided with a regular contact person.” |  |  |
| “I **must** have the option to undergo several surgical treatments during my stay on ward (e.g., neovagina and breast augmentation).” |  |  |
| “Healthcare professionals involved in my treatment **must** compare notes as part of integrative healthcare provision.” |  |  |
| “I **must** be asked by the psychotherapist how appointments with other healthcare professionals involved in the treatment had gone.” |  |  |

Effects of trans healthcare centres on trans healthcare provision

| **I am afraid that interdisciplinary THC centres will…** | **Likert scale 1-6: *I strongly disagree* to *I strongly agree*** |
| --- | --- |
| Monopolise trans healthcare (i.e. that there won’t be any other option to get treatment outside of that one trans healthcare centre). |  |
| Prevent me from being able to choose where I go for trans-related treatment. |  |
| Result in THC professionals expecting me to undergo a certain number of trans-related treatments (e.g., hormone treatment and surgeries). |  |
| Result in my not being able to influence what type of treatment I will get (e.g., regarding different types of medication as part of hormone treatment). |  |
| Lead to my being confronted with ever-changing THC professionals during one single treatment. |  |

1. **Which additional concerns do you have? (OPEN ANSWER)**

Psychotherapeutic counselling

1. **In my view, it is helpful to receive psychotherapeutic counselling during the transition process^[[1]](#footnote-2)^.**
2. Yes
3. No
   1. **Filter:** NO. Why not?
   2. **Filter:** YES. In my view, it is helpful if I can access psychotherapeutic counselling in a trans healthcare centre.
      1. **Filter:** YES. How many sessions with the counsellor per quarter (3 months) would be helpful for you as psychotherapeutic support during your transition?
         - 1. 1 session
           2. 1-3 sessions
           3. More than 3 sessions
           4. I don’t know
      2. **Filter:** YES. In my view, it is helpful to be offered the following non-localised options for psychotherapeutic counselling (i.e. counselling sessions where you don’t go and see the counsellor in person).
         - - Video-supported phone call
           - Phone call
           - Email
           - Other, please specify: (OPEN ANSWER)

**Filter**: YES. Why is it helpful to be able to access psychotherapeutic non-localised counselling?

- - 1. **Filter:** YES. In my view, it is helpful to be visited by a/my psychotherapist on ward **shortly BEFORE** my surgery.
       - - Yes
         - No
         - N/A
    2. **Filter:** YES. In my view, it is helpful to be visited by a/my psychotherapist on ward **shortly AFTER** my surgery.
       - - Yes
         - No
         - N/A
       1. **Filter:** YES. How frequent should such visits be **shortly AFTER** your surgery? (OPEN ANSWER)

General psychotherapeutic treatment

1. **In my view, it is helpful to have the opportunity in the context of my psychotherapeutic counselling during my transition^[[2]](#footnote-3)^ to address issues that are not directly connected to my trans identity (e.g., a depression).**
2. **Filter:** YES. How many sessions per quarter (3 months) with the psychotherapist would be helpful for you in the context of psychotherapy during your transition?
   - - - - 1 session
         - 1-3 sessions
         - More than 3 sessions
         - I don’t know
3. **Filter:** YES. In my view, it is helpful to be offered the following non-localised options for general psychotherapy.
   - - - - Video-supported phone call
         - Phone call
         - Email
         - Other, please specify: (OPEN ANSWER)
4. **Filter**: YES. Why is it helpful to be able to access non-localised general psychotherapy?

Research

In order to develop the quality of trans healthcare centres further, research on trans and health is frequently conducted in this context. Individuals that access treatment in these centres therefore are asked regularly whether they would like to participate in research projects. These research projects seek to, for example, improve treatment quality for trans individuals.

1. **It is important to me that in trans healthcare centres there is research conducted regarding trans and health.** (Likert scale 1-6)
2. **I am willing to participate in research on trans and health in the context of a trans healthcare centre.** (Likert scale 1-6)
3. **Filter:** APPROVAL. It is important to me that I and other trans individuals are being informed about the research results.
4. **Filter:** DISAPPROVAL. Why would you not like to participate in research on trans and health in the context of a trans healthcare centre? (OPEN ANSWER)

Peer contact and support groups

Some clinics facilitate contact between individuals currently treated in the clinic and former patients who offer support to current patients on a voluntary basis (peer support). In this context, experiences regarding treatment, the trans healthcare centre, or effects of hormone treatment can be exchanged.

1. **In my view, it is helpful to come into contact with trans individuals who are familiar with the respective trans healthcare centre and treatment as part of such a co-operation.** (Likert scale 1-6)
2. **Filter:** APPROVAL. Concerning which aspects of trans healthcare would peer support be helpful for you? (OPEN ANSWER)
3. **Filter:** DISAPPROVAL. Why is peer support not helpful for you? (OPEN ANSWER)
4. **In my view, it is helpful to have the following type of co-operation between a trans healthcare centre and local support groups:**
   - - - - Irregular contact
         - Regular contact
         - Support group representatives act in an advisory capacity
         - Support groups and THC centre should not co-operate
         - Other, please specify: OPEN ANSWER
         - I don’t know
5. **Filter:** APPROVAL. Why? (OPEN ANSWER)
6. **Filter:** DISAPPROVAL. Why not? (OPEN ANSWER)

Feedback and complaints

1. **In my view, it is helpful to have a contact person for potential feedback (both praise and criticism) during my treatment.**(Likert scale 1-6)
2. **Filter:** APPROVAL. For me the following contact option is helpful. [PLEASE SELECT ONE OPTION]. We understand that you might prefer more than one way of establishing initial contact. However, we would like to ask you to indicate which option you prefer most.
   - - Email
     - Phone call
     - Online form (anonymous)
     - Online form (with name)
     - Face-to-face conversation
     - Other (OPEN ANSWER)
     - I don’t know
3. **In my view, it is helpful to have a contact person for potential feedback (both praise and criticism) after my treatment.** (Likert scale 1-6)
4. **Filter:** APPROVAL. For me the following contact option is helpful. [PLEASE SELECT ONE OPTION].

We understand that you might prefer more than one way of establishing initial contact. However, we would like to ask you to indicate which option you prefer most.

- - - Email
    - Phone call
    - Online form (anonymous)
    - Online form (with name)
    - Face-to-face conversation with my psychotherapist
    - Other (OPEN ANSWER)
    - I don’t know

Patient involvement in decision-making processes

| Please indicate how much you agree or disagree with the following statements. | **Likert scale 1-6: *I strongly disagree* to *I strongly agree*** |
| --- | --- |
| I want to decide how much I am going to be involved in decisions for or against specific treatment options. |  |
| I know exactly which treatments I want to undergo and just want the respective healthcare professional to implement them as quick as possible. |  |
| Sometimes it is convenient to just let go of all responsibility during medical treatment. |  |
| Ultimately I want to decide on my medical treatment, after having looked in great depth at the opinion of the healthcare professional involved. |  |
| I want the healthcare professional involved in my treatment to make the ultimate decision on my medical treatment, after having consulted me on my opinion. |  |
| I want the healthcare professionals and myself to share responsibility in deciding which treatment might be best for me. |  |
| I want to leave all decisions regarding my medical treatment to the healthcare professional involved in my treatment. |  |
| I want to decide which medical treatment I undergo (e.g., hormone treatment, genital surgery). |  |

1. **It is important to me to be involved in decisions regarding my hormone treatment.**(Likert scale 1-6)
2. **Filter:** APPROVAL. Regarding which aspects exactly? (please select all that apply)
   - - Selection and dosage
     - Start of treatment
     - Changes in the effectiveness during in the course of treatment
     - How to handle unwanted effects and adverse effects
     - Other: OPEN ANSWER
     - I don’t know
3. **In my view, it is helpful to be able to access appointments with my endocrinologist over the course of my hormone treatment.**(Likert scale 1-6)
4. **Filter:** APPROVAL. For me the following contact option is helpful. [PLEASE SELECT ONE OPTION].
   - - Face-to-face contact
     - Contact over the phone
     - Other: OPEN ANSWER
     - I don’t know
5. **Filter:** APPROVAL. For me the following intensity of contact is helpful.
   - - Whenever necessary
     - Regularly, e.g. once every month
     - Regularly, e.g. once every quarter (3 months)
     - Other: OPEN ANSWER
     - I don’t know
6. **In my view, it is helpful to receive information and advice in a trans healthcare centre regarding hormone medication that wasn’t prescribed by the healthcare professional involved in my treatment.** (Likert scale 1-6)

Follow-up care

The following questions cover the time directly after treatment as well as life in the longer term after treatment.

1. **In my view, it is helpful to have several follow-up appointments with the surgeons involved after my surgery/surgeries. [PLEASE SELECT ONE OPTION]**
   1. Yes, pre-arranged
   2. Yes, whenever necessary
   3. No
   4. I don’t know
2. **In** my **opinion the following ambulant offers for follow-up care and support are helpful after my surgical treatment: (ambulant: only in the clinic/ surgery temporarily, as an out-patient): [MULTIPLE ANSWERS POSSIBLE]**
   1. Post-surgical psychotherapy
   2. Post- surgical physiotherapy
   3. Assistance in dealing with surgery results (e.g. with using a dilator, with care for scars)
   4. Other: OPEN ANSWER
   5. I don’t know
3. **In my view, it is helpful to be able to access rehabilitation offers after my surgical treatment(s) as a day patient or inpatient (day patient: spending the days at the clinic, and sleeping at home. Inpatient: spending both days and nights at the clinic).** (Likert scale 1-6)
4. **Filter:** APPROVAL. For me a co-operation between the trans healthcare centre and a rehabilitation clinic would be helpful.
5. **For me it is helpful if the following aspects of general healthcare are offered in a trans healthcare centre [MULTIPLE ANSWERS POSSIBLE]:**
   1. Cancer screening
   2. Bone density
   3. Fertility options
   4. Support regarding contact with health insurance companies
   5. Other, please specify: OPEN ANSWER
   6. I don’t know
6. In **my view, it is helpful, if general healthcare is offered in a trans healthcare centre which takes special needs trans individuals might have into account.**
7. Yes
8. No
9. I don’t know
10. Filter: YES. Why?
11. Filter: NO. Why not?

1. Social and physical changes towards the preferred gender. [↑](#footnote-ref-2)
2. Social and physical changes towards the preferred gender. [↑](#footnote-ref-3)
